# Purification of silicon powder by the formation of thin porous layer followed byphoto-thermal annealing

**DOI:** 10.1186/1556-276X-7-444

**Published:** 2012-08-08

**Authors:** Marouan Khalifa, Messaoud Hajji, Hatem Ezzaouia

**Affiliations:** 1 Laboratoire de Photovoltaïque, Centre des Recherches et des Technologies de l’Energie (CRTEn), Technopôle de Borj-Cédria BP 95, Hammam-Lif, 2050, Tunisia; 2 Institut Supérieur d’Electronique et de Communication de Sfax (ISECS), Route Menzel Chaker Km 0.5 BP 868, Sfax, 3018, Tunisia

**Keywords:** Silicon powder, Porous silicon, Vapor-etching, Thermal annealing, Gettering, ICP-AES

## Abstract

Porous silicon has been prepared using a vapor-etching based technique on a commercial silicon powder. Strong visible emission was observed in all samples. Obtained silicon powder with a thin porous layer at the surface was subjected to a photo-thermal annealing at different temperatures under oxygen atmosphere followed by a chemical treatment. Inductively coupled plasma atomic emission spectrometry results indicate that silicon purity is improved from 99.1% to 99.994% after annealing at 900°C.

## Background

Porous silicon (PSi) is a nano-structured material that can be obtained by electrochemical [1], stain etching [2,3], or vapor phase etching of silicon wafers [4,5]. The main advantages of stain etching and vapor etching methods, if compared with electrochemical one, are their simplicity and capability to produce large area porous silicon layers. Porous silicon elaborated by different methods is extensively used in photovoltaic applications as an antireflection coating or as a gettering layer due to its large specific surface and chemical reactivity. Gettering of impurities by the formation of a thin porous silicon layer followed by a thermal annealing in a nitrogen, oxygen, or SiCl_4_ atmosphere has been used [6,7]. It was found that porous layer play a crucial role in the gettering process. Porous silicon was also used in combination with phosphorous or aluminum gettering of unwanted impurity in silicon [8-10].

In this paper, we present the possibility of gettering impurities from commercial silicon powder (SPw) by photo-thermal annealing in oxygen atmosphere using a thin porous silicon layer on the surface of silicon grains. The gettering effect was studied using the inductively coupled plasma atomic emission spectrometry (ICP-AES).

## Methods

Porous silicon thin layer was formed by exposing silicon powder to the vapor of an acid mixture composed of HF/HNO_3_ with 1:3 composition volume. The etching time was varied from 2 to 20 min. The obtained material was rinsed in deionized water, dried, and then analyzed using Fourier transform infrared spectroscopy (FTIR) and photoluminescence (PL) measurements.

The silicon powder with thin porous silicon layer is then subjected to a photo-thermal annealing stage under oxygen atmosphere in the aim to remove the unwanted impurities. The annealing temperature was varied from 700°C to 900°C for a fixed duration of 1 h. After this purification step, silicon powder was chemically cleaned in NaOH (1 M) solution in order to remove the porous layer and rinsed in deionized water. The purification process was evaluated by ICP-AES method.

## Results and discussions

**Figure 1:**
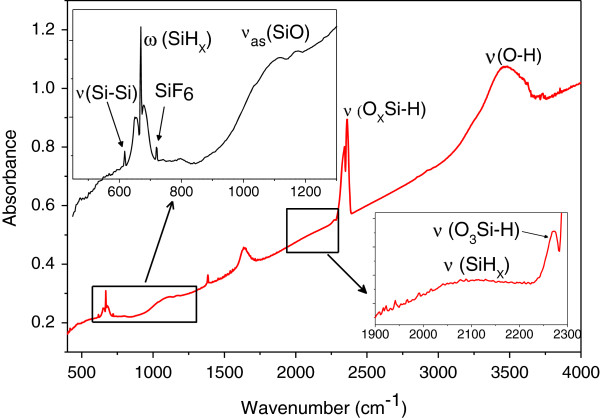
FTIR spectrum of silicon powder with a thin porous layer.

In order to study the effect of vapor etching treatment on the chemical composition of silicon surface, FTIR spectra were recorded. FTIR spectrum of the silicon powder after acid vapor-etching is depicted in Figure 1. Observed FTIR bands are located at around 600 to 750 cm^−l^ (wagging modes), 800 to 1,000 cm^−1^ (bending modes), and 2,050 to 2,200 cm^−1^ (stretching modes) associated with Si-H_*n*_ (*n* ≥ 1) bondings. The band at 1,000 to 1,300 cm^−l^ corresponds to the stretching modes of the Si-O-Si bonds in the SiO_*x*_. In this band, the peak at 1,100 cm^−1^ represents the Si-O-Si anti-symmetric stretches, and the peak at 1,170 cm^−1^ corresponds to the Si-O vibration bands [3]. A sharp absorption band at 2,200 to 2,500 cm^−1^ is observed, and it can be attributed to the O_*x*_-Si-H groups [3,5,11]. The weak absorption band located at 610 to 620 cm^−1^ corresponds to Si-Si stretching modes [4]. The broad band from 3,050 to 3,850 cm^−1^ corresponds to O-H stretching modes in SiOH groups and H_2_O [11], and the 1,630 cm^−1^ band is due to O-H scissor bending vibration in water [12]. SiOH group formation is due to the reaction of SiF_*x*_ with water [13,14].

**Figure 2:**
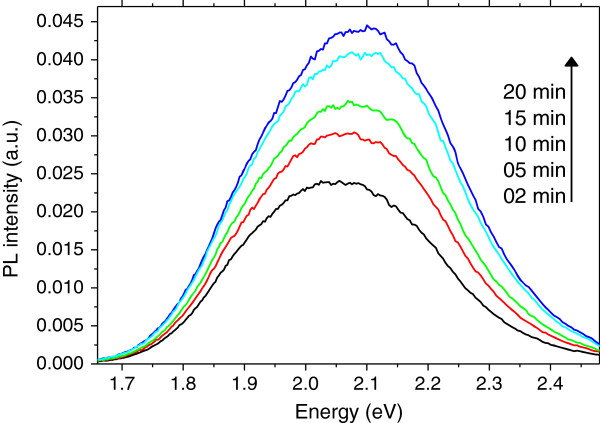
**PL spectra of PSi powder for different etching times.** Curves: blue, 20 min; light blue, 15 min; green, 10 min; red, 5 min; and black, 2 min.

**Figure 3:**
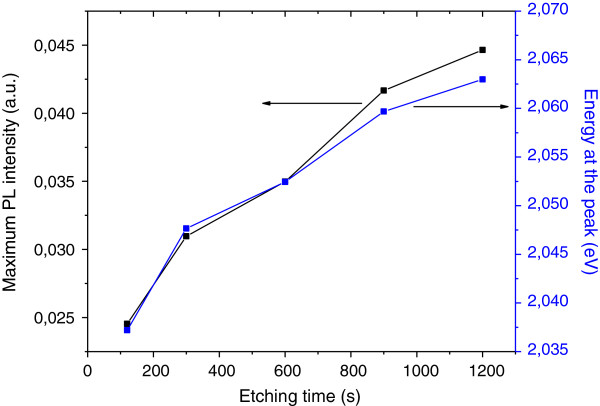
Evolution of the maximum of PL intensity and energy at the peak with etching time.

The photoluminescence properties were obtained using an unfocused argon-ion laser with an excitation wavelength of 488 nm at room temperature. Figure 2 shows the PL spectra of porous silicon elaborated on silicon powder by acid vapor etching method during different etching times. It was found that both the PL intensity and the energy at the peak increase by increasing the etching time (Figure 3). The increase in the intensity is due to an enhancement in the luminescent center density that can be associated to an increase in the thickness of the porous layer [5]. The shift to high energy values is generally attributed to a decrease in the luminescent crystallite size. In this case, the energy at the peak is around 2.05 eV which is higher than values obtained for porous silicon elaborated by the same method on silicon wafers. This shift to higher energies can be attributed to the oxidation of the porous layer. The oxidation leads to the substitution of Si-H bonds by Si-O-H groups and the formation of a Si-SiO_*x*_ interface, resulting in a blue shift of the PL peak [9,15]. This result is in agreement with the FTIR results that show a very weak band located at 2,050 to 2,200 cm^−1^ corresponding to SiH_*n*_ bonds.

**Figure 4:**
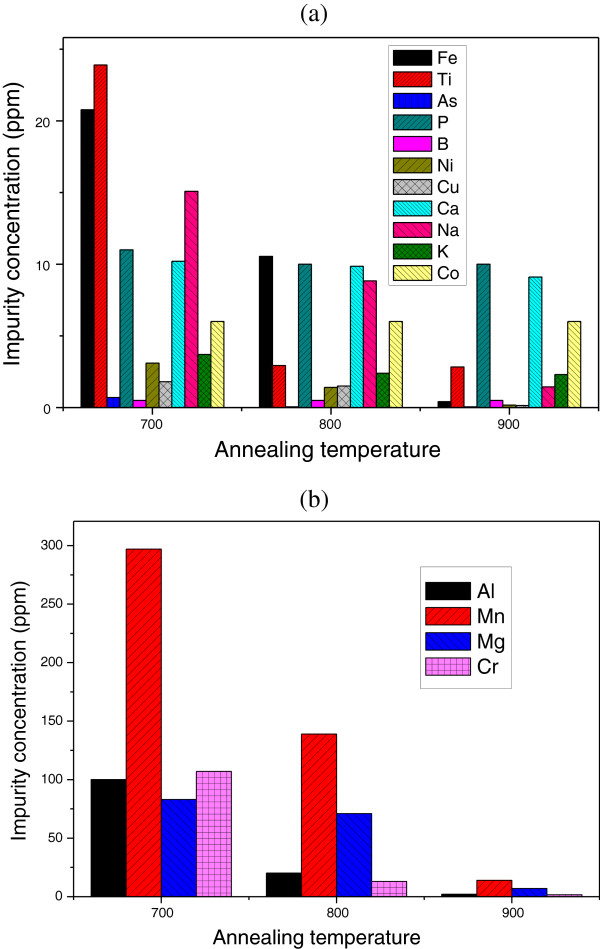
**Evolution of the concentration with the annealing temperature for impurities.** Impurity concentration less than 25 ppm (**a**) and those with a concentration higher than 25 ppm (**b**).

After the discussion of porous silicon properties, we will focus our interest in this section to the study of its gettering effect when subjected to a photo-thermal annealing stage at different temperatures. Table 1 resumes the concentrations of different impurities present in silicon powder before and after purification steps. Depending on their concentrations obtained after the thermal annealing at 700°C, the impurities are divided into two groups: the first contains impurities with a concentration less than 25 ppm, and the second contains those with higher concentrations. The evolution of the concentration with the annealing temperature for two groups is presented in Figure 4a,b. Results show an important decrease in the impurity concentrations after the formation of a porous layer followed by thermal annealing. This reduction is as important as the annealing temperature is higher. After the thermal annealing at 900°C, about 99.991% of Fe, 99.03% of Al, 99.26% of Cr, and 98.24% of Mn were removed; and the purity of silicon powder increases from 99.1% (for the untreated powder) to about 99.995%.

**Table 1:** Impurity concentrations (ppm) before (SPw) and after thermal annealing

	**SPw (ref.)**	**700°C**	**800°C**	**900°C**
Fe	5,100	20.77	10.54	0.41
Al	2,200	100.15	20.17	2.13
Ti	421	23.9	2.93	2.83
As	2	0.7	<0.05	<0.05
P	16	11	10	10
B	0.7	<0.05	<0.05	<0.05
Ni	5.6	3.1	1.4	0.17
Cu	5.5	1.8	1.5	0.14
Ca	98.1	10.21	9.85	9.1
Na	38	15.09	8.84	1.44
Mn	793	297	139	13.9
Mg	55	83	71	7
K	20	3.7	2.4	2.3
Cr	230	107	13	1.7
Co	7	6	6	6
Total (%)	0.9	0.07	0.03	0.01
Purity (%)	99.10081	99.93166	99.97034	99.99489

Annealing temperatures are 700°C, 800°C, and 900°C of silicon powder with a thin porous layer.

The improvement of the silicon powder purity is attributed to the migration of unwanted impurities from the volume of silicon grains composing the powder to the PS layer, at the surface of the grain, where they can be easily removed by chemical etching [6,7]. This gettering technique is an easy and efficient way to improve the quality of silicon intended for solar grade silicon production from metallurgical grade silicon powder.

## Conclusion

This work presents an easy, inexpensive, and efficient method for the removal of impurities from silicon powder. Obtained results show that the purity of silicon powder can be improved from 99.1% for the untreated powder to 99.995% after annealing at 900°C under oxygen atmosphere. This method is very interesting for the production of solar grade silicon from metallurgical grade silicon powder.

## Competing interests

The authors declare that they have no competing interests.

## Authors’ contributions

MK carried out all the experiments and data analysis, and participated in the interpretation of the results. MH co-supervised the work, participated in the concept of the study, and wrote the manuscript. HE supervised the work and revised the manuscript. All authors read and approved the final manuscript.

## Authors’ information

MK is a Ph.D. student in the Laboratory for Photovoltaic, CRTEn. MH is an assistant professor in the ISECS and a researcher in the Laboratory for Photovoltaic, CRTEn. EH is a professor in the Laboratory for Photovoltaic, CRTEn.
